# Supplementary material for: Facile Synthesis of Functionalized Phenoxy Quinolines: Antibacterial Activities against ESBL Producing Escherichia coli and MRSA, Docking Studies, and Structural Features Determination through Computational Approach
Source: Molecules. 2022 Jun 10;27(12):3732. doi: 10.3390/molecules27123732 (PMC9230019; doi:10.3390/molecules27123732)
Supplement: Supplementary file 1 [file molecules-27-03732-s001.zip › molecules-1689790-supplementary.pdf]

Supplementary Data

Facile Synthesis of Functionalized Phenoxy Quinolines: Antibacterial Activities against ESBL Producing *Escherichia coli* and MRSA, Docking Studies, and Structural Features Determination through Computational Approach

Mahwish Arshad [1], Nasir Rasool [1,*], Muhammad Usman Qamar [2], Syed Adnan Ali Shah [3,4] and Zainul Amiruddin Zakaria [5,*]

Department of Chemistry, Government College, University Faisalabad, Faisalabad 38000, Pakistan;
mahwishnadeem001@gmail.com (M.A.)

2 Department of Microbiology, Government College University, Faisalabad 38000, Pakistan; musmanqamar@gcuf.edu.pk (M.U.Q.)

3 Faculty of Pharmacy, Universiti Teknologi MARA Cawangan Selangor Kampus Puncak Alam,
42300 Bandar Puncak Alam, Selangor, Malaysia; syedadnan@uitm.edu.my (S.A.A.S)

4 Atta-ur-Rahman Institute for Natural Product Discovery (AuRIns), Universiti Teknologi MARA Cawangan Selangor Kampus Puncak Alam, 42300 Bandar Puncak Alam, Selangor, Malaysia

5 Department of Biomedical Sciences, Faculty of Medicine and Health Sciences, Universiti Malaysia Sabah, Jalan UMS, Kota Kinabalu 88400, Sabah, Malaysia

* Correspondence: nasirrasool@gcuf.edu.pk (N.R.); zaz@ums.edu.my (Z.A.Z.); Tel.: +92-332-749-1790 (N.R.); Fax: +92-41-920-1032 (N.R.); +60-19-2117090 (Z.A.Z.)

Table of Contents

Table S1: Comparison of experimental and theoretical ^{1}H-NMR data of compound **3b** S3

Table S2: Comparison of experimental and theoretical ^{1}H-NMR data of compound **3c**............. S4

Table S3: Comparison of experimental and theoretical ^{1}H-NMR data of compound **3d** S5

Table S4: Comparison of experimental and theoretical ^{1}H-NMR data of compound **3e** S6

Table S5: Comparison of experimental and theoretical ^{1}H-NMR data of compound **3f**............. S7

Table S6: Comparison of experimental and theoretical ^{1}H-NMR data of compound **3g** S8

Table S7: Comparison of experimental and theoretical ^{1}H-NMR data of compound **3h** S9

Figure S1: MIC of different compounds against ESBL producing *E. coli*.................................... S10

Figure S2: MIC of different compounds against MRSA... S10

Figure S3: MBC of ESBL producing *E. coli* and MRSA S11

Table S1: Experimental and Theoretical Calculated ^{1}H-NMR of compound **3b.**

Carbon No.	Carbon Type	Experimental ^{1}H-NMR (δ, ppm)	Calculated ^{1}H-NMR (δ, ppm)	Δδ, ppm
1	N	-	-	-
2	CH	8.44	9.32	-0.88
3	CH	6.79	6.02	0.77
4	C	-	-	-
5	CH	8.14	8.86	-0.72
6	C	-	-	-
7	CH	7.67	7.93	-0.26
8	CH	8.05	8.25	-0.20
1'	C	-	-	-
2'	CH	6.86	7.37	-0.51
3'	CH	6.73	7.59	-0.86
4'	C	-	-	-
5'	CH	6.86	7.59	-0.73
6'	CH	6.73	7.37	-0.64
Mean Absolute Error (MAE) = 0.23				
Root Mean Square Error (RMSE) = 0.40				

Table S2: Experimental and Theoretical Calculated ^{1}H-NMR of compound **3c.**

**Carbon No.**	**Carbon Type**	**Experimental ^{1}H-NMR (δ, ppm)**	**Calculated ^{1}H-NMR (δ, ppm)**	**Δδ, ppm**
1	N			
2	CH	**8.68**	8.74	-0.06
3	CH	7.49	7.16	0.33
4	C			
5	CH	8.57	8.62	-0.05
6	C			
7	CH	**7.81**	7.91	-0.10
8	CH	8.02	8.16	-0.14
1'	N			
2'	C			
3'	CH	**8.41**	7.46	0.95
4'	CH	7.07	8.16	-1.09
5'	CH	**7.07**	7.38	-0.31
6'	CH	6.89	8.24	-1.35

Mean Absolute Error (MAE) = 0.23

Root Mean Square Error (RMSE) = 0.40

Table S3: Experimental and Theoretical Calculated ^{1}H-NMR of compound **3d.**

Carbon No.	Carbon Type	Experimental ^{1}H-NMR (δ, ppm)	Calculated ^{1}H-NMR (δ, ppm)	Δδ, ppm
1	N			
2	CH	**8.21**	8.58	-0.37
3	CH	7.49	6.38	1.11
4	C			
5	CH	8.07	8.71	-0.64
6	C			
7	CH	**7.65**	7.91	-0.26
8	CH	8.02	8.12	-0.10
1'	S			
2'	CH	**7.95**	7.22	0.73
3'	C			
4'	CH	7.15	7.07	0.08
5'	CH	**7.35**	7.43	-0.08
1	N			

Mean Absolute Error (MAE) = 0.23

Root Mean Square Error (RMSE) = 0.40

Table S4: Experimental and Theoretical Calculated ^{1}H-NMR of compound **3e.**

**Carbon No.**	**Carbon Type**	**Experimental ^{1}H-NMR (δ, ppm)**	**Calculated ^{1}H-NMR (δ, ppm)**	**Δδ, ppm**
1	N			
2	CH	**8.45**	8.49	-0.04
3	CH	6.98	5.99	0.99
4	C			
5	CH	8.12	8.63	-0.51
6	C			
7	CH	**7.67**	7.83	-0.16
8	CH	8.05	8.09	-0.04
1'	C			
2'	CH	**6.82**	7.16	-0.34
3'	C			
4'	CH	6.82	7.39	-0.57
5'	C			
6'	CH	6.82	7.16	-0.34
Mean Absolute Error (MAE) = 0.10<br>Root Mean Square Error (RMSE) = 0.25				

Table S5: Experimental and Theoretical Calculated ^{1}H-NMR of compound **3f.**

Carbon No.	Carbon Type	Experimental ^{1}H-NMR (δ, ppm)	Calculated ^{1}H-NMR (δ, ppm)	Δδ, ppm
1	N			
2	CH	**8.45**	8.48	-0.03
3	CH	6.98	6.00	0.98
4	C	-		
5	CH	8.13	8.64	-0.51
6	C			
7	CH	**7.67**	7.83	-0.16
8	CH	8.05	8.08	-0.03
1'	C			
2'	CH	**6.92**	7.42	-0.50
3'	C			
4'	C			
5'	CH	**6.82**	7.38	-0.56
6'	CH	6.7	7.23	-0.53
Mean Absolute Error (MAE) = 0.14				
Root Mean Square Error (RMSE) = 0.29				

Table S6: Experimental and Theoretical Calculated ^{1}H-NMR of compound **3g.**

Carbon No.	Carbon Type	Experimental ^{1}H-NMR (δ, ppm)	Calculated ^{1}H-NMR (δ, ppm)	Δδ, ppm
1	N			
2	CH	**8.47**	8.44	0.03
3	CH	7.06	5.94	1.12
4	C			
5	CH	8.13	8.67	-0.54
6	C			
7	CH	**7.67**	7.82	-0.15
8	CH	8.05	8.07	-0.02
1'	C			
2'	CH	**6.86**	7.32	-0.46
3'	CH	**6.74**	7.90	-1.16
4'	C			
5'	CH	**6.74**	7.90	-1.16
6'	CH	6.86	7.32	-0.46
Mean Absolute Error (MAE) = 0.17<br>Root Mean Square Error (RMSE) = 0.37				

Table S7: Experimental and Theoretical Calculated ^{1}H-NMR of compound **3h.**

Carbon No.	Carbon Type	Experimental ^{1}H-NMR (δ, ppm)	Calculated ^{1}H-NMR (δ, ppm)	Δδ, ppm
1	N			
2	CH	**8.47**	8.45	0.02
3	CH	7.67	5.93	1.74
4	C			
5	CH	8.14	8.68	-0.54
6	C			
7	CH	**7.81**	7.83	-0.02
8	CH	8.05	8.08	-0.03
1'	C			
2'	CH	**6.85**	7.37	-0.52
3'	CH	**6.96**	8.34	-1.38
4'	C			
5'	CH	**6.85**	8.50	-1.65
6'	CH	6.96	7.46	-0.50
Mean Absolute Error (MAE) = 0.27<br>Root Mean Square Error (RMSE) = 0.59				

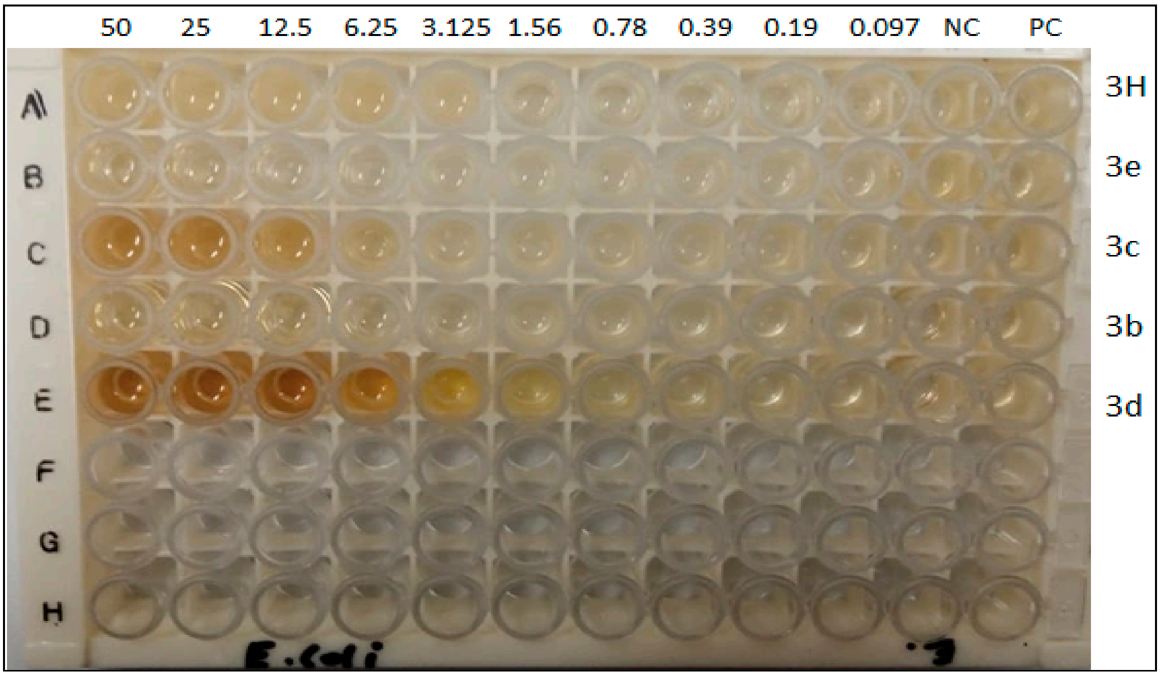

Figure S1: MIC of different compounds against ESBL producing E. coli.

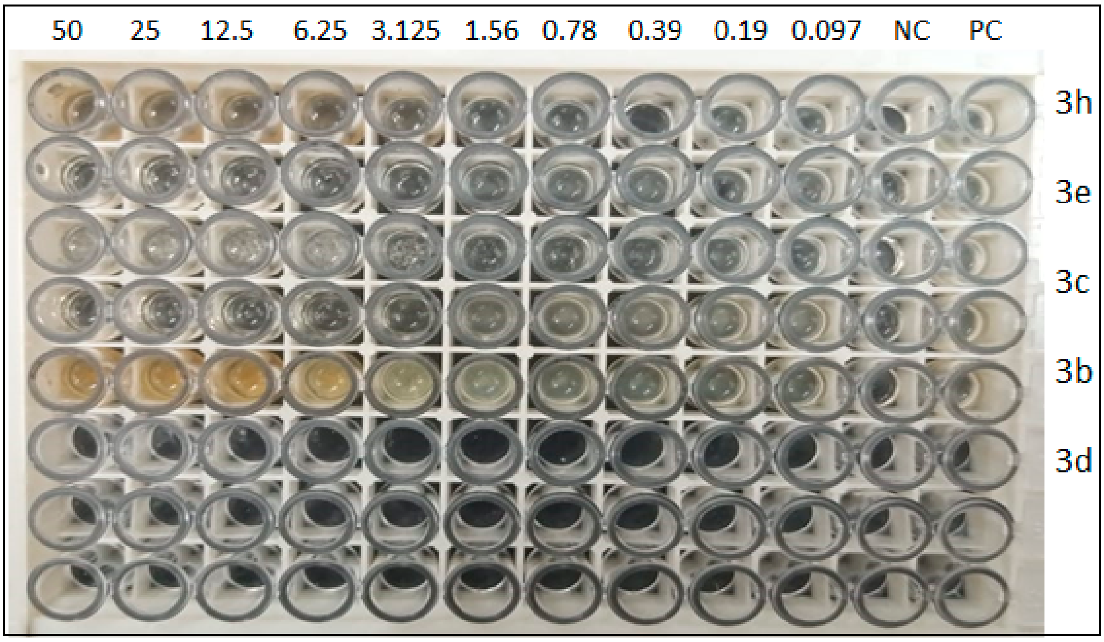

Figure S2: MIC of different compounds against MRSA.

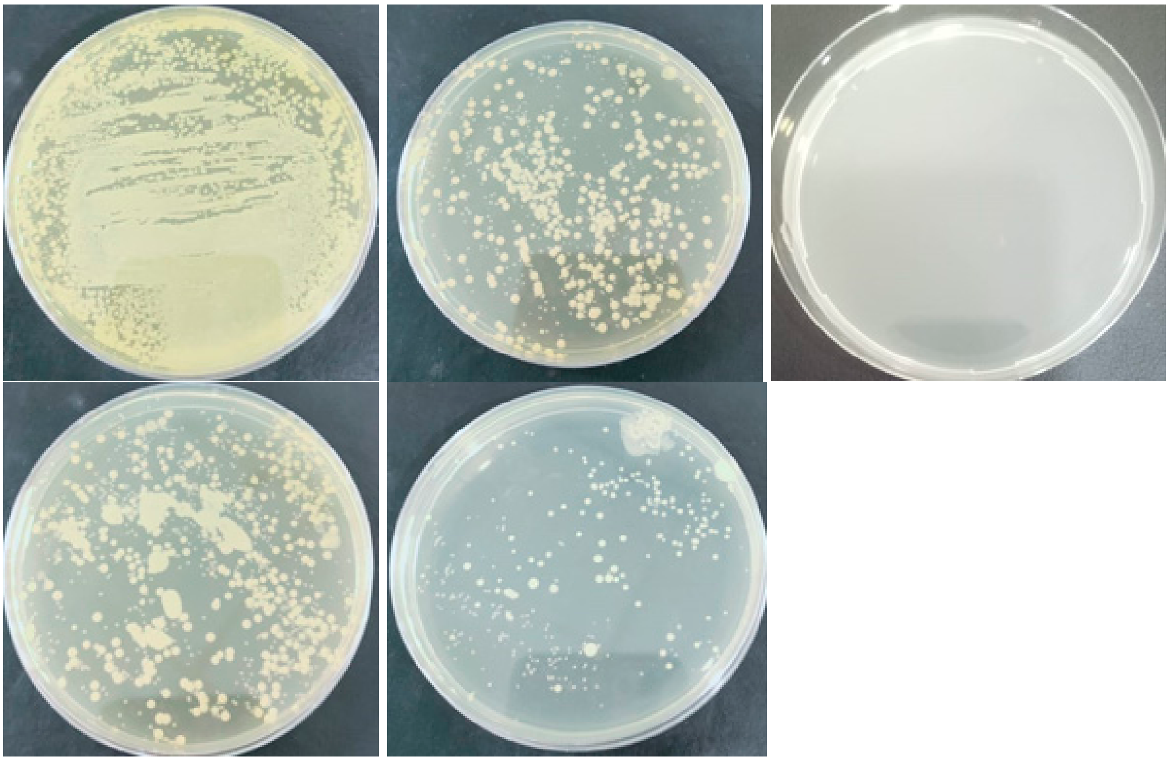

Figure S3: MBC of ESBL producing E. coli and MRSA.
